# Geological Voyage

**Published:** 1841-05

**Authors:** 


### GEOLOGICAL VOYAGE.

Dr. D. D. Owen, Geologist of Indiana, has fitted out a boat in Cincinnati, and commenced a voyage from that city to the mouth of the Ohio river, for the collection of geological specimens. Starting from the oldest rock formation of the Ohio valley, and floating over the outcroppings, to the east, of many successive formations of limestone, shale and sandstone, the results of his exploration cannot fail to be interesting. He is desirous of the co-operation of all who live near the river or up its tributary streams; and we hope our readers in the region through which he passes, will make to him a liberal contribution of specimens,—noting in all cases, with accuracy, their localities; and when they send organic remains, crystallized salts, or ores, transmitting, also, specimens of the rocks in which they are found. Dr. Owen is one of your very best geologists, and deserves every kind of encouragement in his scientific enterprize. D.

